# Iodine content of dietary salt at household level and associated factors using Iodometric titration methods in Dera District, Northwest Ethiopia

**DOI:** 10.1186/s40795-017-0203-x

**Published:** 2017-11-28

**Authors:** Zelalem Alamrew Anteneh, Melese Engidayehu, Gedefaw Abeje

**Affiliations:** 1 School Public Health, College of Medicine and Health Sciences, Bahir Dar University, Bahir Dar, Ethiopia; 2 Health and health related logistics and institutions inspector, Ethiopian food, medicine and health care control authority, Northwest branch of Ethiopia, Bihar Dar, Ethiopia

**Keywords:** Iodine content, Ethiopia, Titration method

## Abstract

**Background:**

In developing countries most of the edible salts have insufficient iodine content; the problem is worse in Africa. Only 15.4% of the Ethiopian population was using adequately iodized salt. Several factors affect iodine content of edible salt including poor handling practices. The existing evidence isn’t sufficient to detail the specific factors at the household level. Therefore, the aim of this study was to determine the iodine content of edible salt and identify factors associated with salt iodine content in Dera District, Northwest Ethiopia.

**Methods:**

A community-based cross-sectional study was conducted among 1194 households. A multi-stage sampling technique was used to select the households, and data were collected using the interview. A 50 g salt sample was collected from each selected household and was shipped to the Ethiopian Food, Medicine and Health Care administration and Control Authority (EFMHACA) laboratory center for iodine level analysis. The samples were analyzed using titration method. Data were entered into EPI-INFO and analyzed in SPSS.

**Results:**

Out of 1194 salt samples collected, 57.4% had iodine content in the range 15 ppm to 59.42 ppm. Salt stored in closed containers was more likely to have better iodine content compared to salt stored with open containers (AOR = 1.7, 95% CI: 1.24–2.42). Salt samples stored in dry places were 1.5 times more likely to retain iodine compared to samples stored near to heat/fire or in a moist area (AOR = 1.5, 95% CI: 1.03–2.14). Similarly, salt samples stored for less than 2 months were more likely to have adequate iodine level compared to samples stored for over 2 months (AOR = 1.6, 95% CI: 1.12–2.29).

Salt samples obtained from household heads attended primary education (AOR = 1.5, 95% CI: 1.05–2.26), high school (AOR = 1.7, 95% CI: 1.05–2.64), and University (AOR = 2.8, 95% CI: 1.06–5.62) were more likely to have adequate iodine content in edible salt compared to whose didn’t attend formal education.

**Conclusions:**

Nearly three out of five salt samples had enough iodine content. However, this level is low compared to the WHO recommendation (90%). The age, educational status of head of the household, duration of salt storage, use of cover to store salt and knowledge of household heads were associated with an iodine content of salt. Therefore, use of cover and proper storage of edible salt should be encouraged; improving the educational status of the community is essential the edible salt to retain its iodine content at the household level.

**Electronic supplementary material:**

The online version of this article (10.1186/s40795-017-0203-x) contains supplementary material, which is available to authorized users.

## Background

Iodine is an essential micronutrient for humans required in a very small amount [1, 2]. The daily recommended amount of iodine for normal function of thyroid gland is 150–200 μg/l for adults, 90–120 μg/l for children and 250 μg/l for pregnant & lactating mothers [3–5]. World health organization (WHO) recommends that the median iodine urinary level need to be within the range (100–199 μg/l) to ensure adequate iodine content in salt and other sources of iodine in the diet. Or iodized salt containing 15 to 40 ppm of iodine at the household level regarded as adequately iodized [6].

Iodization of salt is first line measure to prevent and control iodine deficiency disorders. The Ethiopian Council of Ministers passed salt legislation in February 2011; according to this regulation, every salt for human consumption need to be iodized, and any iodized salt for human consumption shall conform to the standards for iodized salt set by the appropriate authorit [7].

Iodine Deficiency (ID) is the most common preventable cause of intellectual impairment. Globally, 241 million population are estimated to have insufficient iodine intakes. The problem is worse in Southeast Asia and Africa, where 76 million and 58 million populations have inadequate iodine intake respectively [8]. According to the Ethiopian National Nutrient survey report, 47.5% of school children had urinary iodine levels less than 100 μg/L. The median Urinary iodine level in non-pregnant women of reproductive age was 96.8 μg/L; about 51.8% of women had urinary iodine levels less than 100 μg/L. The report also showed that only 26% of the total households were getting more than 15 ppm iodine in salt [9]. Similarly, Ethiopian Demographic and Health Survey (EDHS) 2011 indecated that about 66 million persons were unprotected from iodine deficiency, and only 15% of households had access to iodized salt [10].

A study in Sudan showed that 85.6% of the population obtained 13.7 mg of iodine in salt [11]. In Ghana, 5% of the population consumes salt without iodine and 19.4% of the population consumes below the country’s recommended level [12]. Another study in Haiti showed that among salt samples tested for iodine content, 86% of the table salts labeled as “iodized” were found with no iodine at all [13]. A similar study conducted in India showed that out of the total salt samples collected, 13.2% had zero mg, 4.1% 1-5 mg and 17.4% 6-15 mg of iodine per 1 kg of salt [14].

A study conducted in Benishagul Gumze region, western Ethiopia, pointed out that 27.0% and 52.9% of the households were consuming salt with zero, and between zero to 15 ppm iodine content respectively [15]. And a study done in Hawassa town, Ethiopia showed that all salt samples tested for iodine had iodine concentration below the recommended level [16]. Another study done in Laelay Maychew district, Northern Ethiopia showed that 19.2% and 38% of the households were consuming salt with zero, and less than 15 mg/kg of iodine content [17]. A similar study conducted in Lay Armachiho rural district showed that 37.5% of salt samples reported no iodine in it and 32.8% contained 1–15 mg iodine per kg of salt [18].

Improper storage, poor handling practices and buying non iodized salt are some of the cause for low iodine content in salt [19–21]. Studies showed that there are several causes for low iodine content of edible salt at household level; a study conducted in India showed that iodine content in salt was reduced due to personal and environmental causes from its production site to the consumer level [22].

In Ghana, salt handling practice was the main cause for loss of iodine content in salt [12].

A study conducted in Southern Ethiopia identified age, education level, poor knowledge and poor handling practices as important predictors of iodine content of edible salt of households [16].

Although Ethiopia is enforcening the universal salt legislation to increase the content of iodine in edible salt, there is a limited evidence on the level of iodine content of salt and factors that affect its content at household level. Therefore, this study was designed to determine the iodine content of salt and identify factors associated with its content in households in Dera district.

## Methods

### Study design and period

A community-based cross-sectional study was done in December 2015.The study performed in South Gondar zone Dera district, found between 1500 and 1800 m above sea level, 612kms from Addis Ababa (the capital of Ethiopia) in the Northwest and 42kms from Bahir-Dar (the capital city of Amhara region).

The district comprises 32 kebeles (the smallest administrative units in Ethiopia), 87% of the population living in the distrct have access to health care; there were 10 health centers and36 health posts in the district.

### Study population and variables

All households of Dera district living in the selected kebeles were eligible for the study. Households and individuals in the household who were responsible (the woman) in food preparation were the study units.

### Sample size and sampling procedures

A single population proportion formula used using EPINFO. The following assumptions were used: 95% confidence interval, assuming that 36.3% of salt samples is adequately iodized, 80% power, 1.61 odds ratio, 5% margin of error. Adding 10% non-response rate and using design effect of 2 the final sample size was 1241.

Multistage sampling procedure was used. From the total of 32 kebeles in the District, five were selected randomly. The calculated sample size was allocated into the selected Kebeles based on the number of households they had. Households were selected using systematic sampling strategy. The salt samples were collected in each household and were analyzed in the Amhara region FMHACA laboratory and the concentration of iodine was measured in titration method. In this study, the salt collected from the households was considered adequately iodized if the iodine level in the salt is from 15 to 40 mg of iodine per kg of salt [6].

### Data collection procedures

Data were collected using interview administrated questionnaire. The questionnaire was adapted from reviewed literature [16–18, 23] (Additional file 1). The data were collected by 11 data collectors who had similar data collection experiences in the rural community. Two days training was given to data collectors on the purpose of the study, data collection techniques, and on ethical issues in research. They were also trained on how to collect the salt samples. A pretest was conducted in Zenzelima Kebele (rural kebele of Bahir Dar city administration), which was not included the main study. Necessary changes were made to the questionnaire before the actual data collection. A 50 g salt sample was collected carefully from the selected households with an airtight plastic bag.

An information sheet with the date of sample collection, code of the sample, and type of salt, packing material, batch number if, date of production, expiry date, name and signature of data collector was attached to the salt sample. Salt samples were packed and sent from the data collectors to the supervisors daily. The supervisors sent the salt samples to the investigators the next day to the laboratory (North-west branch of EFMHACA) in Bahir Dar city (where titration analysis was done). There are different tests to assess the level of iodine in salt. In this study, we used the titrimetric titration method which is 85.4% sensitive and 71.25% specific to detect the level of iodine in salt. The equipments needed, the chemical and test procedures are annexed (Additional file 2).

### Data analysis

The collected data were checked for errors and completeness on daily basis. Data were entered into EPI- Info version 7 and exported to SPSS version 20 for analysis.

Frequencies and percentages were done to see the magnitude of events in the study. Bivariate logistic regression analysis was computed to test whether there is an association between Iodine content of salt and selected independent variables.

Independent variables with a significance level less than or equal to 0.20 in the bivariate logistic regression analysis were entered to the multivariable logistic regression analysis. The multivariable logistic regression model was built with backward elimination.

## Results

### Socio-demographic characteristics

Data were collected from 1194 households (96.2% response rate). The mean and standard deviation of the age of respondents was 35.19 ± 12.67 years. The majorities (90%) of the respondents were females.

Two hundred and fifty (20.9%) respondents could not read and write, and 358 (30%) engaged in adult education. Most respondents (97.8%) were Amhara in Ethnicity, and 1047 (87.7%) were Orthodox Christians in religion (Table 1).

**Table 1:** Socio-demographic characteristics of respondents in Dera District, North West Ethiopia, Dec 2015

Variables(*N* = 1194)	Frequency	Percent
Age of the respondent		
18-29 years	507	42.5
30–44 years	391	32.7
≥ 45 years	296	24.8
Wealth Index		
Poor	395	33.3
Medium	401	33.6
Rich	398	33.1
Occupation of the respondent		
Housewife	942	78.9
Government employee	60	2.7
Merchant	89	7.5
Daily Laborer	32	2.7
Others (Beggars)	25	2.1
Family Size		
< 5	1046	87.6
5 or more	148	12.4
Marital Status		
Married	785	65.7
Single	218	18.3
Divorced	112	9.4
Separate	15	1.3
Widowed	64	5.4

### Iodine content and handling practices of edble salt at household level in Dera District

In this study, iodine content of edible salt varied from 0.00 to 59.42 ppm (ppm) with a mean and standard deviation of 20.79 and 11.56 ppm respectively. Out of the total salt samples tested for iodine content, 685 (57.4%) had acceptable iodine content between (15–59.42 ppm) based on the WHOs cut of points. However, 44 (3.6%) salt samples had no Iodine at all, and the remaining 40.6% salt samples contained less than 15 mg of iodine /kg of salt.

**Fig. 1:**
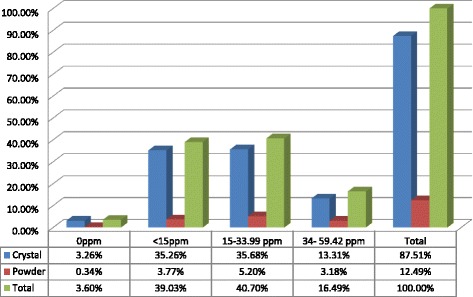
The Iodine contents in the crystal and powder forms of salt in Dera District, Northwest Ethiopia, Dec. 2015

Regarding the salt the households use, 87.51% used the crystal form and the rest were using powdered and packed. Out of the total salt samples with adequate iodine content, the majority 71.8% were in crystal form (See Fig. 1).

About 376 (31.5%) respondents reported that they used iodized salt properly; 453 (37.9%) of them used for about 2–4 years. Five hundred and fifty (46.1%) households reported that iodized salt is accessible for them to buy within a reasonable distance.

In addition, 1003(84%) of the households bought their iodized salt from their usual market, the only handful of them bought from village shops.

Seven hundred and ninety-seven (66.8%) of households place their salt in the covered container. When tested for iodine content, 77.5% of the salt samples from this household were found adequately iodized.

Of the total study households, 939 (78.6%) reported that they store their salt in a dry area, 86.1% of salt samples obtained from these households were found adequately iodized. Similarly, 997 (83.5%) of the households reported that they stored their salt for less than 2 months. Among those who store salt for less than 2 months, 89.6% of salt samples were adequately iodized (See Table 2).

**Table 2:** Iodine content of edible salt and handling practices in Dera District, North West Ethiopia, Dec-2015

Variables	Frequency	percent
Salt buy from		
Usual market	1003	84
Village shop	191	16
Type of salt used		
Crystal	1045	87.51
Powder	149	12.49
Salt leveling observable by data collector		
Yes	137	11.5
No	1057	88.5
Salt storage place		
Dry	939	78.6
Moist area	254	21.4
Storage or storage time in house		
**<** 2 months	997	83.5
2 or more months	197	16.7
Salt exposed to sun/heat		
Yes	28	2.3
No	1166	97.3
Iodine content in salt		
≥ 15 ppm	683	57.4
< 15 ppm	511	42.6

*ppm* parts per million

### Factors associated with edible salt iodine content

According to the multivariable logistic regression analysis, salt samples from respondents aged 18 to 29 and 30 to 44 years were more likely to have adequate iodine level compared to those whose age was above 45 years (AOR = 2.2, 95% CI: 1.60–3.13) and (AOR = 2.2, 95% CI: 1.6–3.16) respectively. Salt samples from households who stored salt in a closed containers were 1.7 times more likely to have adequate iodine content compared to salt samples obtained from households who stored in an open containers (AOR = 1.7, 95% CI: 1.24–2.42). The study also showed that salt samples from households who store salt in dry place were 1.5 times more likely to have adequate iodine content compared to households who place salt in moist area (AOR = 1.5, 95% CI: 1.03–2.14).

Similarly, salt samples taken from households who stored salt for less than 2 months were 1.6 times more likely to have adequate iodine content compared to those salt samples taken from households who stored for over 2 months (AOR = 1.6, 95% CI: 1.12–2.29) (See Table 3).

**Table 3:** Factors associated with iodine content in salt at household level in Dera district, Dec 2015

Variables	Iodine content		COR (95% CI)	AOR (95% CI)
	<15 ppm	≥15 ppm		
Age of respondents				
18–29	180	341	3.3(2.47–4.53)	2.2(1.60–3.13)
30–44	151	240	2.8(2.04–3.85)	2.2(1.60–3.16)
45 & above	180	102	1	1
Storage time in house				
< 2 months	383	614	3.0(2.16–4.01)	1.6(1.12–2.29)
2 and above months	128	69	1	1
Educational of respondents				
Unable to read & write	155	95	1	1
Able read & Write	168	190	2(1.43–2.76)	1.28(0.89–1.84)
Primary	114	216	3.1(2.22–4.40)	1.5(1.05–2.26)
Secondary	56	134	4.0(2.68–6.00)	1.7(1.06–2.64)
Higher	16	50	7(3.62–13.50)	2.8(1.39–5.62)
Use Cover for their salt				
Yes	266	531	3.2(2.45–4.04)	1.7(1.24–2.31)
No	243	154	1	1
Salt Storage Place				
Dry Area	350	589	3.4(2.63–4.34)	1.5(1.03–2.14)
Near Heat/fire area	161	94	1	1
Knowledge respondents				
Good Knowledge	362	564	2(1.48–2.57)	1.7(1.27–2.31)
Poor knowledge	149	119	1	1

## Discussions

The aim of this study was to find out the iodine content of edible salt at household level and the factors that may affect iodine content of edible salt in the rural community of Dera district. The study identified that three out of five (57.4%) salt samples had acceptable iodine content based on the WHO recommended level. According to the WHO and International Council for Control of Iodine Deficiency (ICCIDD) standard, elimination of Iodine Deficiency Disease (IDD) will be possible if over 90% of the households consume adequately iodized salt [24].

The percentages of edible salt with acceptable iodine at household level in this study was higher than a similar study conducted in 2014 in the Northwest region of Ethiopia [18].

Similarly, our finding was higher than studies conducted in Kenya in 2015 26.2% [25], and in Senegal 10% [26], Sudan 14.4% [11] and Haiti 11% [13] in 2012.

The possible reason for higher proportion of iodine content in our study may be because Ethiopia has made a major effort to stimulate and improve iodine status under the national salt iodization strategy for the last few years. The government of Ethiopia dedicated group ministry of health (FMHACA) to work up on iodization and other essential activities in health; the ministry then has banned sale of non-iodized salt for direct human consumption throughout the country in 2012 [27]. In addition, this study was undertaken after the Ethiopian government enforces salt legislation for mandatory salt iodization which was ratified and approved by Council of Ministers. Regular follow up and monitoring might cause awareness and the rate of salt iodization may be increased; this might raised availability & accessibility of iodized salt at household level. However, the proportion of households with adequate iodine content in salt in this study was lower compared to studies conducted in Nigeria 95% [28], Ghana 75.6% [12] India and Tajikistan(64%, 71% respectively) [29, 30].

The possible reason for this discrepancy may be the difference in technology and institutional factors to refine iodize salt, lack of enforcement in the legislation, and quality-controlled iodization technology at the production site, awareness of community, effective transport channels, correct labeling, packaging and storage.

This study identified that 44 (3.6%) of salt samples didn’t contain iodine at all; which entails people consumed salt without iodine. Similar findings were observed in studies conducted in Ghana (5%) and India (13.2%) in 2012 where the salt samples were found with zero iodine content at household level [31, 32]. However, the results of the current study are much better than studies conducted in Lay Armachiho and Benishagulgumze in Ethiopia by 2014, where the percentages of zero level iodine content in edible salt were 23.5% and 27% respectively [15, 18].

Age of respondents was significant predictors of iodine content of salt. Respondents of younger age (<44 years) were more than two times more likely have had adequately iodized salt at home compared to those 45 and older. This finding was consistent with studies in Gondar [23] and Kenya [25] where salt samples collected from young aged respondents were more likely to contain an adequate level of iodine in salt than samples collected from respondents of older age. This might be linked to the exposure to different social media and other information sources on iodized salt. As age advances exposure to different media likely to decrease.

Educational level of respondents was associated with the availability of adequately iodized salt in the household. A similar finding was found in study conducted in Kenya, where the educational status of the respondent was associated with an adequate level of iodine in edible salt in the household [25].

The findings of the current study also identified that the storage place of iodized salt was an important factor for the availability of adequately iodized salt in the household. Storing edible salt in dry and enclosed containers at household level helps the salt to retain its iodine content. Our finding was supported by other similar studies conducted in Gondar and Lay Armachiho district [17, 23], where availability of adequately iodized salt were high in those storing their salt in dry area and closed containers than those storing their salt near fire/heat or in moist area and open containers. This may be due to the fact that if salt stored in humid condition and stay for longer period, it attracts moisture and becomes wet, carrying the iodide part to the bottom of the container. At hot condition, salt can release surface moisture, and this may cause an iodine loss by its volatility if the container is opened. This study identified the level of iodine in salt at households only. However, salt may lose its iodine content at production, transport and storage sites. Therefore, this study fails to show the specific situation where iodine content of the salt is lost.

## Conclusions

The availability of adequately iodized salt at household level in the district is lower than the WHO recommended level.

We also found out that age and educational status of the household heads, salt storage time, and storage area were associated with the presence of adequately iodized salt at household level.

Therefore, education should be promoted. Concerned organizations including the regional education bureau should encourage women’s education to improve iodine content of salt at household level.

We encourage the households to put their iodized salt in dry place in a closed container to preserve the iodine content of the salt. What is more, we discourage to store iodized salt more than 2 months, as the salt loses its iodine content.

Finally, further study is needed to identify the place of iodine loss from the salt; is it at place of manufacture, during transport or in the shop.

## Additional files

Additional file 1: The English Version Questionnaires. (DOCX 30 kb)

Additional file 2: Equipment, chemicals used and procedures followed. (DOCX 22 kb)
