# Supplementary material for: Iodine content of dietary salt at household level and associated factors using Iodometric titration methods in Dera District, Northwest Ethiopia
Source: BMC Nutr. 2017 Nov 28;3:83. doi: 10.1186/s40795-017-0203-x (PMC7050691; doi:10.1186/s40795-017-0203-x)
Supplement: Supplementary file 1 — The English Version Questionnaires. (DOCX 30 kb) [file 40795_2017_203_MOESM1_ESM.docx]

Questions

| **No** | | **Questions** | | | **Possible responses** | | **Skip Resp.** |
| --- | --- | --- | --- | --- | --- | --- | --- |
| **Part: 1 Socio demographic Factors** | | | | | | |  |
| 101 | Responsibility of respondents | | 1. 1. Mother, 2. Father, 3. G/mother, 4. other | | | |  |
| 102 | The respondent relation with the mother? | | 1. 1. Father 2. Mother 3. Grand mother   4. Relatives 5. Others | | | |  |
| 103 | Sex of the respondent | | 1. Female 2. Male | | | |  |
| 104 | Age of the respondent | | --------------- | | | |  |
| 105 | What is your Religion? | | 1. Orthodox, 2. Muslim 3. Protestant, 4. Catholic 5. Others/specify__ | | | |  |
| 106 | Ethnicity of respondents | | 1. Amhara 2. Tigray 3. Other | | | |  |
| 107 | Current marital status | | 1. Married 2. Divorced 3. Single 4. Widowed, 5. Separate | | | |  |
| 108 | Educational status of the households(if the respondent is no wife) | | 1. Unable to read and write, 2. Able read and write 3. Primary (1-8 grade) 4. Secondary (9-12 grade) 5. Higher education | | | |  |
| 109 | Educational status of Mother | | 1. Unable to read and write, 2. Able read and write 3. Primary (1-8 grade) 4. Secondary (9-12 grade) 5. Higher education | | | |  |
| 110 | Educational status of Father | | 1. Unable to read and write 2. Able to read and write 3. Primary (1-8 grade) 4. Secondary (9-12 grade) 5. Higher education | | | |  |
| 111 | Occupation of Father | | 1. Daily laborer, 2. Merchant 3. Govt. Employee 4. . Other/specify | | | |  |
| 112 | Occupation of mother | | 1. Housewife 2. Daly laborer 3. Merchant  4. Govt. Employee 5. Other/specify __ | | | |  |
| 113 | How many family members live in the house? | | --------------- | | | |  |
| 114 | Is/are there pregnant woman /women in the HH? | | 1. Yes 2. No | | | |  |
| 115 | If yes, how many? | | ----------- | | | |  |
| 116 | Is/are there lactating woman/women in the HH? | | 1. Yes 2. No | | | |  |
| 117 | If yes, how many? | | -------------- | | | |  |
| 118 | Is/are there child/children <3 years in the HH? | | 1. Yes 2. No | | | |  |
| 119 | If yes, how many? | | --------------------- | | | |  |
|  | **Wealth indicators** | |  | | | |  |
| 120 | Do you have a radio in the house? | | 1. 1. Yes 2. No | | | |  |
| 121 | Do you have Mobile phone in the house? | | 1. Yes 2. No | | | |  |
| 122 | Do you have Television in the house? | | 1. Yes 2. No | | | |  |
| 123 | How much is your monthly income in Birr? | | ----------------- | | | |  |
| 124 | Do you have your Own house? | | 1. 1. Yes 2. No | | | |  |
| 125 | What type of flooring do you have? | | 1. 1. Mud, 2. Cement 2. 3. Other, specify--------------- | | | |  |
| 126 | Do you have the following in your home? | | 1. Caw and ox, 2. Horse & donkey  3. Ship ፣goat, Hen 4. Nothing | | | |  |
|  | **PART 2*.* Knowledge questions** | | | | | |  |
| 201 | Have you ever heard about iodized salt before? | | | 1. 1. Yes 2. No | | | If No go to Q.206 |
| 202 | What is the source of information about iodized salt? More than one answer is possible | | | 1. Health workers 2. Radio 3. Television   4. Friends/ relatives 5. Others (specify)---- | | |  |
| 203 | Do you know the importance of iodized salt intake for human health? | | | 1. 1. Yes 2. 2. No | | | If no, go to Q205 |
| 204 | Why intake of iodized salt is important? More than one answer is possible  (Don’t read the option circle only what they say) | | | 1. To cure goiter,2.To remain healthy   3.To prevent IDD, 4.To grow well  5.Better than other salt, 6. I Don’t know   1. Others (specify)--------------- | | |  |
| 205 | What are the consequences?  (Don’t read the option circle only what they say). More than one answer is possible | | | 1. Goiter 2. Mental retardation 3. Growth retardation  4. Stillbirth/abortion 5. Loss of learning ability   1. Others (specify)----------- | | |  |
| 206 | Do you think any salt contains iodine? | | | 1. 1. Yes 2. No | | |  |
| 207 | Do you know iodized salt has the label of iodine or expiry date on their container? | | | 1. 1. Yes 2. No | | |  |
| 208 | Do you know the characteristics and standards of iodized salt | | | 1. Yes No | | |  |
| 209 | Do you think if the storage of iodized salt kept near heat/fire has no problem? | | | 1. 1. Yes 2. No 3. I don’t know | | |  |
| 210 | Do you think iodine content reduces when iodized salt is not stored in enclosed containers? | | | 1. 1. Yes 2. No 3. I don’t know | | |  |
| 211 | Do you think your edible salt contains iodine? | | | 1. Yes No | | |  |
|  | **Part 3 Practice Questions** | | | | | | |
| 301 | For how long do you use iodized salt in your house in year | | | | | 1. 1. <2 yrs 2. 2-4yrs, 3. Above 4 yrs 5. I don’t know |  |
| 302 | Where do you buy iodized salt from | | | | | 1. Village shop 2. Market day 3. Others (specify)-------------- |  |
| 303 | Approximate distance in km(single trip) to get iodized salt | | | | | 1. ------------------------------- |  |
| 304 | What type of salt do you bought your iodized salt? | | | | | 1) Crystalline salt   1. 2) Powdered and packed salt 3. other |  |
| 305 | Do you check the level of iodine before you buy the salt | | | | | 1.Yes 2.No |  |
| 306 | Salt leveling Observable By Interviewer | | | | | - - - 1. Yes 2.No |  |
| 307 | Do you use a cover for your salt container? | | | | | 1. Yes 2. No |  |
| 308 | Do you expose your salt to sunlight/heat? | | | | | 1. 1. Yes 2. No |  |
| 39 | Do you wash your salt to remove impurities from the salt? | | | | | 1. 1. Yes 2. No |  |
| 310 | Where is the salt storage place?  ( Observe the area) | | | | | 1. 1. Dry area 2. Nearest the fire/heat or moist area |  |
| 311 | For how long use the iodized salt after you bought from the market?  ( storage time) | | | | | 1.<2 months   1. 2 and above months |  |
| 312 | Salt sample No | | | | | Type of salt | Iodine content |

***THANK YOU FOR YOUR TIME***
