# Supplementary material for: Iodine content of dietary salt at household level and associated factors using Iodometric titration methods in Dera District, Northwest Ethiopia
Source: BMC Nutr. 2017 Nov 28;3:83. doi: 10.1186/s40795-017-0203-x (PMC7050691; doi:10.1186/s40795-017-0203-x)
Supplement: Supplementary file 2 — Equipment, chemicals used and procedures followed. (DOCX 22 kb) [file 40795_2017_203_MOESM2_ESM.docx]

## Equipment and Chemicals

### Equipment

Chemical balance/Electronic balance/Electrical balance, reagent bottles with stoppers à 250 ml, 500 ml & 1000 ml, Measuring cylinder with stopper- 50 ml, Wash bottle, 500 ml and Gas burner, Conical flask with stopper, 100 ml, Glass or plastic funnel , Burette, 50 ml , Burette, stand Pipette – 25 ml and Glass stirring rod**,** A closed box, cupboard or drawer to keep the conical flask**,** Physical balance (capacity 50 – 100 gms)

### Chemicals

Sodium thiosulphate, (2Na2 S2 03 5H2O)**,** Concentrated sulfuric acid, (H2SO4) Potassium iodide (KI) **,** Soluble chemical starch**,** Potassium Iodate

All the chemicals should be analytical grade and double distilled water to be used which should be free of iodine and other contaminants.

### Preparation of reagents

1.0.005 Normal Sodium thiosulphate (Na2S203) Dissolve 1.24 g sodium thiosulphate crystals (Na2S203.5Hz0) in 1 L boiled, double-distilled water. This volume is sufficient for testing 200 salt samples. Store in a cool, dark place. Properly stored, the solution can be kept for a few months. Standardize the sodium thiosulphate solution every three months using standard potassium iodate solution.

2. N Sulphuric acid (H2SO4) To 90 ml double-distilled water add 6.0 ml concentrated sulphuric acid (H2SO4) slowly. Add boiled, double-distilled water to make 100 ml. This volume is sufficient for 100 salt samples. Store in a cool dark place. The solution may be kept indefinitely. Caution: To avoid violent and dangerous reaction always add the acid to water, never water to acid! Stir the solution while adding.

3. Potassium iodide (KI, AR) Dissolve 100 g KI in 1 Liter of double-distilled water. This volume is sufficient for testing 200 salt samples. Store in a cool, dark place. Properly stored, the solution may be kept for 6 months.

4. Soluble chemical starch Dissolve reagent-grade sodium chloride (NaCI) crystals in 100 ml boiled, double-distilled water. While stirring, add NaCl until no more dissolves. Heat the contents of the beaker till excess salt dissolves. While cooling the NaCl crystals will form on the sides of the beaker. When it is completely cooled, decant the supernatant into a clean bottle. This can be stored for 3 to 4 weeks. Dissolve 1 g of chemical starch in 10 ml boiling double-distilled water. Continue to boil till it completely dissolves. Add the saturated NaCl solution to make 100 ml starch solution. This volume is sufficient for testing 20 salt samples. Prepare fresh starch solution every day since starch solution cannot be stored.

### Laboratory procedure

1. Carefully weigh 10 g of the salt to be tested;

2. Pour the salt into a 50 ml measuring cylinder & lowly add boiled, double-distilled water;

3. Shake to dissolve the salt completely and add more water to make 50 ml;

4. Pour the salt solution (50ml) into a conical flask with stopper;

5. Pipette out 1 ml of 2N sulfuric acid and add this to the salt solution;

6. Pipette out 5 ml of 10% potassium iodide and add this to the salt solution;

7. (Do not pipette acid or KI by mouth!)

8. The solution turns yellow. Close the flask with the stopper and put it in the dark for 10 minutes. A closed box, cupboard or drawer may be used;

9. Pour 0.005N sodium thiosulfate solution into a burette.

10. Adjust the level in the burette to "0"; 19. Note the burette reading.

11. After 10 minutes, take the flask out of the dark box;

12. shaking the flask, titrate the solution in the flask with sodium thiosulfate from the burette.

13. Stop titration as soon as the solution turns pale (becomes very light yellow.

14. Add a few drops (1 to 5 ml) of 1% starch solution to the flask.

15. The solution turns deep purple.

16. Continue titration until the purple coloration disappears and the solution becomes colorless.

17. From the attached table, read the iodine content of the sample in ppm
